# Distinct Intramuscular Extracellular Matrix Protein Responses to Exercise Training in COPD and Healthy Adults and Their Association with Muscle Remodeling

**DOI:** 10.3390/cells14211656

**Published:** 2025-10-22

**Authors:** Davina C. M. Simoes, Efpraxia Kritikaki, Gerasimos Terzis, Ioannis Vogiatzis

**Affiliations:** 1 Faculty of Science and Environment, Northumbria University, Newcastle upon Tyne NE18ST, UK; effie.kritikaki@newcastle.ac.uk; 2 School of Physical Education and Sports Science, National and Kapodistrian University of Athens, 17237 Athens, Greece; gterzis@phed.uoa.gr; 3 Faculty of Health and Wellbeing, Northumbria University, Newcastle upon Tyne NE18ST, UK; ioannis.vogiatzis@northumbria.ac.uk

**Keywords:** ECM, skeletal muscle, exercise-training, pulmonary rehabilitation, COPD, healthy, fibronectin, tenascin C, collagen, SPARC, biglycan

## Abstract

**Highlights:**

**What are the main findings?**

- In COPD, exercise training induced distinct alterations in ECM protein expression related to tissue structure, cell adhesion, myogenesis, and necroptosis.
- These alterations differed from the adaptive remodeling observed in healthy controls.

**What are the implications of the main findings?**

- ECM proteins play a crucial role in regulating muscle fiber remodeling in response to exercise training.
- Impaired ECM remodeling may contribute to the reduced skeletal muscle adaptability seen in COPD patients, potentially limiting the effectiveness of exercise interventions.

**Abstract:**

**Background**: The skeletal muscle extracellular matrix (ECM) is critical for muscle force and the regulation of important physiological processes. A growing body of evidence demonstrates that in aging, altered ECM composition profoundly hinders the capacity for muscle adaptation in response to exercise training. We evaluated the pattern of ECM expression in response to exercise training between healthy young participants and patients with chronic obstructive pulmonary disease (COPD), to provide insight into how normal adaptive processes differ under conditions of chronic disease. **Methods**: Vastus lateralis muscle biopsies from 29 patients (mean ± SD FEV1: 43 ± 16% predicted) and 14 healthy subjects were analyzed before and after an interval exercise training program for myofiber distribution and size. A selection of ECM molecules was quantified using ELISA. **Results**: Compared to healthy participants, patients exhibited a lower capacity to increase myofiber type I distribution (by 4.7 ± 3.4 vs. 1.3 ± 2.2%) and mean fiber cross-sectional area (by 13.6 ± 3.2 vs. 9.1 ± 1.9%). Exercise training induced a diverse protein expression between the two cohorts in ECMs regulating tissue structure (collagens: up-regulated only in COPD), myogenesis (SPARC: up-regulated only in healthy), necroptosis (tenascin C: up-regulated only in COPD), adherence to muscle-cell precursors (Fibronectin: up-regulated only in healthy) and tissue integrity (biglycan: down-regulated only in COPD). **Conclusions**: Impaired ECM remodeling may underlie the reduced exercise training muscle adaptation observed in COPD patients.

## 1. Introduction

Skeletal muscle wasting is a key determinant of quality of life and mortality, whether it is associated with chronic diseases, physiological aging, or deconditioning [1]. An important aspect of muscle wasting is that muscle strength and power decline at a faster rate than muscle mass, resulting in a decrease in the functional quality of muscle [2,3]. Patients with chronic obstructive pulmonary disease (COPD) frequently experience a well-documented loss of peripheral muscle mass and strength. This poses a serious clinical concern, as the reduction in functional muscle mass is associated with poorer recovery following hospitalization and increased mortality rates [2]. While there is a decrease in functional contractile tissue, the relative area occupied by non-contractile intramuscular connective tissue and its extracellular matrix proteins (ECM) proportionally increases [4].

The ECM is a complex network composed of collagens, glycoproteins, and proteoglycans, ECM-associated regulatory molecules, ECM-affiliated proteins, and various secreted factors [5,6]. The composition of the intramuscular ECM plays a crucial role in providing cell support and transmission of contractile force. Additionally, the ECM acts as a reservoir for growth factors, facilitating muscle growth and repair [5]. Despite its critical importance, ECM biomarkers are underinvestigated in studies examining the effects of exercise training, disuse, or aging on muscle adaptation [7]. A well-balanced remodeling of the ECM is conducive to effective muscle adaptation to training, as it regulates cell behavior and influences muscle regeneration and repair capacity by modulating biochemical and mechanical signaling within cells. In addition, adhesive molecules in the ECM act as receptors that respond to environmental changes, triggering adhesion signaling [6,7]. Developing a deeper understanding of the elements within the cell niche can be beneficial for informing the design of therapeutic interventions aimed at enhancing skeletal muscle regeneration throughout an individual’s lifespan.

ECM proteins are continuously synthesized and degraded to replace old and damaged polypeptides [8,9]. While the turnover of some ECM proteins is readily observed, others, such as elastin, aggrecan, and collagen, persist in tissue for decades [9]. This longevity makes them susceptible to oxidative damage, glycation, pathological cross-linking, and degradation by protease [10]. The ECM’s resistance to protein turnover leads to the accumulation of proteins with unfavorable modifications, which increases muscle stiffness and fibrosis, ultimately impairing muscle elasticity and force transmission [10]. Age-dependent changes in ECM composition contribute to impaired muscle repair and decreased force production [11,12].

Exercise training is a powerful stimulus for skeletal muscle regeneration, influencing not only the myogenic processes of muscle but also the adaptive remodeling of the intramuscular ECM [6,7]. In patients with COPD, exercise-based pulmonary rehabilitation is the most effective treatment to counteract at least in part muscle wasting [1]. Although exercise training appears to be the most beneficial strategy for muscle adaptation and regeneration in COPD, the response is limited in these patients [1]. Beyond its effects on the myofiber cross-sectional area (CSA) and the fiber type redistribution, exercise training also induces profound alterations in ECM composition [6]. A growing body of evidence demonstrates that in elderly healthy people and in patients with COPD, altered ECM composition profoundly hinders the capacity for muscle adaptation in response to exercise training [5,11,12,13]. These ECM adaptations depend on the ability of muscle-resident cells to sense stimuli and secrete ECM components. Conversely, the mechanical forces transmitted through the ECM during exercise critically regulate cell signaling, thereby shaping the cell’s capacity to respond to external stimuli [13].

A substantial body of literature has documented the level of expression of ECM molecules in skeletal muscle [6,7]. However, their precise contribution to exercise-induced muscle adaptation remains poorly defined. Moreover, the physiological intramuscular ECM response to training is still underexplored [7]. Previous studies have primarily focused on ECM gene expression [6], which provides only a partial perspective and does not fully capture the functional role of these proteins [6]. Although the expression of ECM molecules is dependent on the type of exercise-training and age, older participants seem to present similar ECM adaptation to young individuals when longer training programs are applied. However, lack of methodological consistency among studies promotes inconsistent differences between studies and hinders the understanding of the role of these ECM proteins in muscle adaptation [6]. To address this gap, the present study investigates young healthy individuals who typically exhibit appropriate remodeling of the intramuscular ECM, a process that facilitates muscle fiber hypertrophy in response to exercise training. By analyzing this population, the study aims to establish a physiological baseline for ECM adaptation against which COPD-related alterations can be compared. Furthermore, the study integrates phenotypic outcomes with analyses of ECM composition in skeletal muscle of healthy young individuals and patients with COPD, providing insight into how normal adaptive processes are altered under conditions of chronic disease. Overall, our findings enhance the understanding of the role of ECM proteins in muscle adaptation in the context of COPD.

## 2. Materials and Methods

### 2.1. Study Population

*Vastus lateralis* muscle biopsies from 29 male clinically stable patients with COPD [1,14] were analyzed. The patients met the following criteria for entry into the study: (1) post-bronchodilator forced expiratory volume in 1 s (FEV_1_) <50% predicted and FEV_1_/forced vital capacity (FVC) <70% without significant post-bronchodilator reversibility (<10% FEV_1_% pred); and (2) optimal medical therapy without regular use of systemic corticosteroids. A group of 14 young, healthy, sedentary male participants [15] was included to provide a reference control for normal ECM muscle adaptation to training [6,7]. The muscle specimens were analyzed at Northumbria University Newcastle (Newcastle upon Tyne, UK) in accordance with the Human Tissue Act 2004 and with approval from Northumbria University Newcastle Ethics Committee (HLSIV220916-V2).

### 2.2. Study Design

COPD patients had completed a 10-week multidisciplinary exercise training program, as previously described [1]. The program involved supervised exercise training (incorporating both resistance exercise and interval aerobic), breathing control and relaxation techniques, methods of clearance of pulmonary secretions, dietary advice, and disease education. Healthy participants completed an 8-week supervised exercise training program also comprising interval aerobic and resistance exercise [13]. Percutaneous biopsies of the right vastus lateralis muscle were performed at mid-thigh (15 cm above the patella) by the Bergstrom technique before (pre-training) and upon completion of an exercise-based PR program (post-training) as detailed elsewhere [1]. The collected muscle specimens were frozen in liquid nitrogen immediately after excision and maintained at −80 °C until analysis for fiber CSA and fiber type characterization [1]. A selection of ECM biomarkers was studied based on their role in myogenesis, as previously detailed in our earlier studies [6,13]. An account of muscle phenotypic characteristics and ECM protein data has been published elsewhere [13,16].

### 2.3. Muscle Biopsy Morphological Analyses

Vastus lateralis muscle percutaneous biopsies were analyzed for phenotypic characteristics as previously described [1]. The analyses of the biopsy samples were performed blindly for fiber type classification, fiber CSA, and capillary/fiber ratio as previously described. For the latter analysis, the number of capillaries in an individual area was divided by the number of fibers found in the corresponding muscle section [13].

### 2.4. Quantitative Real-Time PCR Analysis

RNeasy Fibrous Tissue kit (Qiagen, Manchester, UK) was used to extract total RNA according to the manufacturer’s protocol. The RNA quantification was performed using a NanoDrop spectrophotometer (Thermo Fisher Scientific, Loughborough, UK). Up to 1 μg RNA was used for synthesizing cDNA with SuperScript II reverse transcriptase and RNaseOUT (Invitrogen, Loughborough, UK [13]). Primers (Eurofins MWG, Hamburg, Germany) were designed using Primer3 software (https://primer3.ut.ee/ version 4.1.0, 21 October 2025). Primer sequence is as previously described [13]. Quantitative real-time PCR data are presented as fold changes relative to the housekeeping gene glyceraldehyde 3-phosphate dehydrogenase (GAPDH), estimated using the 2−ΔΔCt method.

The mRNA expression was analyzed for the following ECM molecules: collagen type I heterodimer chains (*COL1A1* and *COL1A2*), collagen type IV (*COL4A1*), fibronectin (*FN*), tenascin C (*TNC*), integrin β1 (*ITGB1*), osteopontin (*SPP1*), secreted protein acidic and rich in cysteine (*SPARC*), decorin (*DCN*), biglycan (*BGN*), paired box 7 (*PAX7*), myoblast determination protein 1 (*MYOD1*). To gain insight as to whether the changes in ECM in COPD muscle are indicative of the capacity of myogenic adaptation, the expression of myogenic regulatory factors (MRFs), including myogenic differentiation 1 (*MYOD1*), paired box 7 (*PAX7*), myostatin (*MSTN*), and integrin B1 (*ITGB1*), was examined [13].

### 2.5. Protein Extraction and Analysis

Muscle tissue samples were lysed using an Omni Rotor Stator homogenizer in RIPA buffer containing cOmplete Mini protease inhibitor cocktail (Roche, Gillingham, UK). The homogenized samples were subjected to end-over-end rotation at 4 °C for 30 min, followed by 10 min centrifugation at 10,000× *g* (4 °C). Total protein in the supernatant was quantified using the Pierce BCA protein assay kit (Thermo Fisher Scientific). Specific ECM protein levels were quantified using ELISA and normalized by 1 μg of total protein. The ELISA kits used were as follows (catalog number/manufacturer): collagen I (ab285250, Abcam, Cambidge, UK), collagen IV (orb562147, Biorbyt, Cambridge, UK), integrin β1 (orb563558, Biorbyt), osteopontin (DOST00, R&D Systems, Abington, UK), fibronectin (DFBN10, R&D Systems), SPARC (DSP00, R&D Systems), tenascin C (EH446RB, Invitrogen), decorin (EHDCN, Invitrogen) and biglycan (EH45RB, Invitrogen) according to the manufacturer’s instructions. The ELISA’s sensitivity and detection range are as previously described [13].

### 2.6. Statistical Analyses

Demographic data on participants and muscle fiber morphology are presented as mean ± SEM. Molecular data are presented as median values with interquartile (25th–75th percentiles) representing post-training changes compared to pre-training (Δ = post-training–pre-training). A two-way repeated-measures ANCOVA (factors: group × time) to assess the effect of exercise training on ECM protein levels between the two groups, with age and baseline ECM protein expression included as covariates to control for potential confounding effects. The Wilcoxon matched pairs signed rank test was used to analyze muscle fiber characteristics between groups and the two-tailed Mann–Whitney test within groups. Correlations between expression levels of target genes/proteins and muscle fiber characteristics were explored using the Spearman correlation coefficient. The statistical significance level was set at *p* < 0.05.

## 3. Results

### 3.1. Myofiber Phenotypic Changes

The demographic characteristics of the participants, along with their lung function metrics at the outset of the study, are summarized in Table 1. An analysis of muscle fiber characteristics between the groups is presented in Table 2. The fractional changes in vastus lateralis from the muscle of COPD patients have been calculated from our previously published data [1,14]. After training, muscle samples obtained from COPD patients demonstrated significantly higher prevalence of type IIa myofibers (54.9%) in comparison to healthy controls (45.2%, *p* = 0.0221). In the control group, this change after training corresponded to a 6.5% increase in the proportion of type IIa fibers (*p* = 0.0208) and a significant decrease in type IIx fibers by 7.8% (*p* = 0.0098). Conversely, the distribution of type I fibers was significantly lower in the COPD group, with 34.3% compared to 44.5% in healthy participants (*p* = 0.0161). Although there was an increase in the proportion of type I fibers in the COPD group, the change was not statistically significant (*p* = 0.4517). Among COPD patients, training resulted in a 5.3% reduction in type IIx fibers (*p* = 0.0001), with no significant changes observed in the proportions of type IIa and type I fibers.

Significant differences were observed in myofiber CSA (μm^2^) between groups. The average CSA for all fiber types, as well as for specific individual fiber types, was significantly lower in the muscle of patients with COPD compared to healthy young individuals. Training significantly increased the myofiber CSA for most fiber types analyzed in both groups (Table 2), except for type I fibers in COPD patients (*p* = 0.0605). Furthermore, the magnitude of improvement in the capillary to fiber ratio was significantly different between groups (Table 2).

### 3.2. Myogenic Regulatory Molecules Are Similarly Induced by Exercise Training in Both Groups

**Figure 1:**
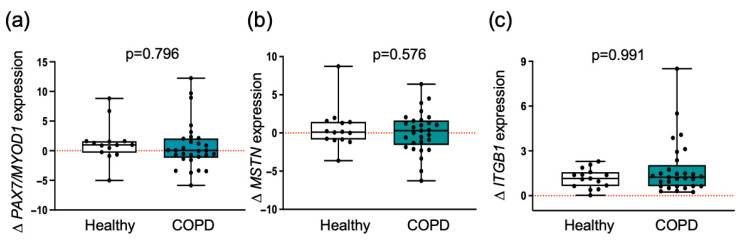
Changes in myogenic regulatory markers following training were similar between groups. Comparison of fold changes in mRNA expression in healthy individuals and patients with COPD is shown. Boxplots show medians (black line) with interquartile ranges for the mRNA expression of (**a**) ratio of *PAX7* to *MYOD1*; (**b**) *MSTN*. Individual participant values are represented as black data points, and (**c**) *ITGB1*. Values between groups were analyzed using a two-way repeated-measures ANCOVA (factors: group × time). The level of significance is indicated in the graphs. The red dotted line indicates the level of no change with exercise training within each group.

PAX7 and MYOD1 are crucial transcription factors involved in regulating the myogenic program. Abnormal interactions between these molecules have been associated with impaired satellite cell function and muscle loss [17,18,19]. Our investigation into the changes in the gene expression ratio of *PAX7* and *MYOD1* following exercise training revealed no significant differences between healthy individuals and patients with COPD (Figure 1). This similarity indicates that exercise training effectively supports normal transcriptional control and elicits an adaptive response in satellite cells in the muscle of COPD patients. These results are also indicative of a comparable level of satellite cell commitment and differentiation in both groups.

**Table 2:** Muscle fiber morphological characteristics of healthy individuals and COPD patients.

	Healthy			COPD		
**Individuals (*n*)**	14			29		
**Fiber type distribution (%)**						
	**Pre-training**	**Post-training**	**∆ (post-pre)**	**Pre-training**	**Post-training**	**∆ (post-pre)**
Type I %	39.8 ± 4.6	44.5 ± 2.9	4.7 ± 3.4	33.0 ± 1.8	34.3 ± 2.6 ^†^	1.3 ± 2.2 ^‡^
Type II %	59.2 ± 5.9	57.6 ± 2.5	−1.5 ± 4.1	65.9 ± 2.4	63.4 ± 2.8	−2.6 ± 2.1
Type IIa %	38.9 ± 2.8	45.2 ± 2.5 ^#^	6.5 ± 2.3	52.0 ± 1.9 ^†^	54.9 ± 2.3 ^†^	3.9 ± 1.8 ^‡^
Type IIx %	20.2 ± 3.1	12.4 ± 1.6 ^#^	−7.8 ± 2.6	15.5 ± 0.7	10.4 ± 0.5 ^#^	−5.3 ± 0.8
**Cross-sectional area (CSA, μm^2^)**						
	**Pre-training**	**Post-training**	**%∆ (post-pre)**	**Pre-training**	**Post-training**	**%∆ (post-pre)**
Mean all fibers	4582 ± 155.6	5214 ± 162.7 ^#^	13.6 ± 3.2	4100 ± 106.9 ^†^	4391 ± 129.3 ^#,†^	9.1 ± 1.9 ^‡^
Type I	3974 ± 146.8	4425 ± 207.8 ^#^	14.0 ± 6.6	4670 ± 171.9 ^†^	5049 ± 241.6	9.2 ± 3.5
Type IIa	5656 ± 205.8	6354 ± 174.9 ^#^	12.0 ± 4.0	4154 ± 138.7 ^†^	4460 ± 170.0 ^#,†^	11.8 ± 3.5
Type IIx	4176 ± 180.2	4813 ± 132.8 ^#^	14.6 ± 3.0	3339 ± 165.2 ^†^	3638 ± 155.6 ^#,†^	10.0 ± 3.7 ^‡^
Capillary/fiber ratio	2.02 ± 0.14	2.13 ± 0.13	13.2 ± 2.5	1.43 ± 0.07 ^†^	1.64 ± 0.07 ^#,†^	16.7 ± 4.3 ^‡^

Data are presented as mean ± SEM. Three experimental replicates per individual muscle sample. *n*: number. † *p* < 0.05 significance level between a COPD group and a healthy control group at the same time-point, # *p* < 0.05 significance level within-group (post-exercise vs. baseline) differences, and ‡ *p* < 0.05 significance of the effect of exercise training between COPD patients and the healthy control group.

Furthermore, we assessed the expression of *MSTN*, whose protein levels are known to negatively regulate muscle growth and size [20]. The results indicated no significant differences in *MSTN* expression between the two groups (Figure 1). Integrin B1 is a transmembrane receptor forming a physical bridge from the ECM and the intracellular cytoskeleton, which plays a critical role in mediating mechanical and biochemical signaling [5]. Muscle from both groups showed a comparable increase in expression of *ITGB1* after training (Figure 1). This finding confirms that training induces similar expression of this integrin in muscle from both groups, which is indicative of similar capacity to transmit contractile and activate muscle growth, repair, and differentiation of satellite cells.

### 3.3. Collagen Differential Expression in COPD Compared to Healthy Individuals

**Figure 2:**
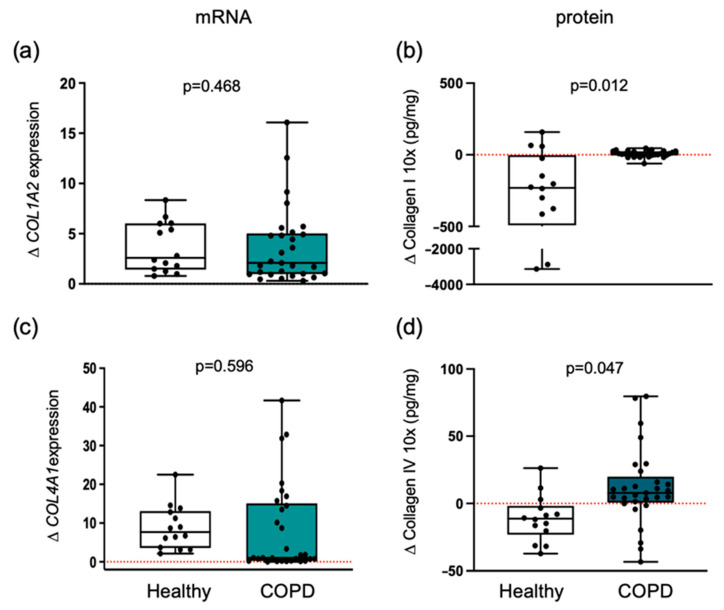
Collagen protein levels were decreased post-training only in healthy individuals. Changes in healthy individuals and patients with COPD are shown. Boxplots depict medians (black line) with interquartile ranges for: (**a**) *COL1A2* mRNA expression and (**b**) collagen type I protein levels; (**c**) *COL4A1* mRNA expression and (**d**) collagen type IV protein levels. Results in plots a and c are fold change, and in plots b and d are absolute concentration changes. Individual participant values are shown as black data points. Values between groups were analyzed using a two-way repeated-measures ANCOVA (factors: group × time). The level of significance is indicated in the graphs. The red dotted line indicates the level of no change with exercise training within each group.

Exercise training induced a differential effect (*p* = 0.012) on collagen type I protein level between COPD patients and healthy individuals (Figure 2). In healthy participants, collagen type I protein levels significantly decreased (*p* = 0.005) from pre-training (median = 644.1 pg/mL; 25th percentile = 142.3, 75th percentile = 1158) to post-training (median = 147.5 pg/mL; 25th percentile = 77.0, 75th percentile = 829.5). In contrast, COPD patients showed no significant change (*p* = 0.080) from pre-training (median = 38.1 pg/mL; 25th percentile = 25.6, 75th percentile = 45.7) to post-training (median = 40.3 pg/mL; 25th percentile = 28.0, 75th percentile = 57.7). However, analysis of mRNA expression for *COL1A1* (Supplementary Figure S1) or *COL1A2* chains revealed no significant differences between groups (Figure 2).

Similar to collagen type I, collagen type IV also exhibited a distinct response (*p* = 0.047) to exercise training in terms of protein levels (Figure 2). In healthy individuals, collagen type IV protein median levels significantly decreased following training (*p* = 0.030), from a pre-training median of 41.7 pg/mL (25th percentile = 30.9, 75th percentile = 62.5) to a post-training median of 32.9 pg/mL (25th percentile = 26.9, 75th percentile = 45.7). In contrast, patients with COPD demonstrated a significant increase (*p* = 0.009), with median values rising from 37.2 pg/mL (25th percentile = 28.1, 75th percentile = 53.1) pre-training to 48.12 pg/mL (25th percentile = 32.8, 75th percentile = 67.6) post-training.

Training-induced changes in collagen IV protein levels were inversely correlated with alteration in mean CSA across all participants (r_S_ = −0.517, *p* = 0.020). Moreover, a significant inverse correlation was observed between changes in collagen IV protein levels and type IIx fibers (r_S_ = −0.561, *p* = 0.021) across all participants.

*COL4A1* gene expression was increased after training in both groups, without a significant difference in the magnitude of change between groups (Figure 2).

### 3.4. Myogenic Proteoglycans Are Downregulated in COPD Patients

**Figure 3:**
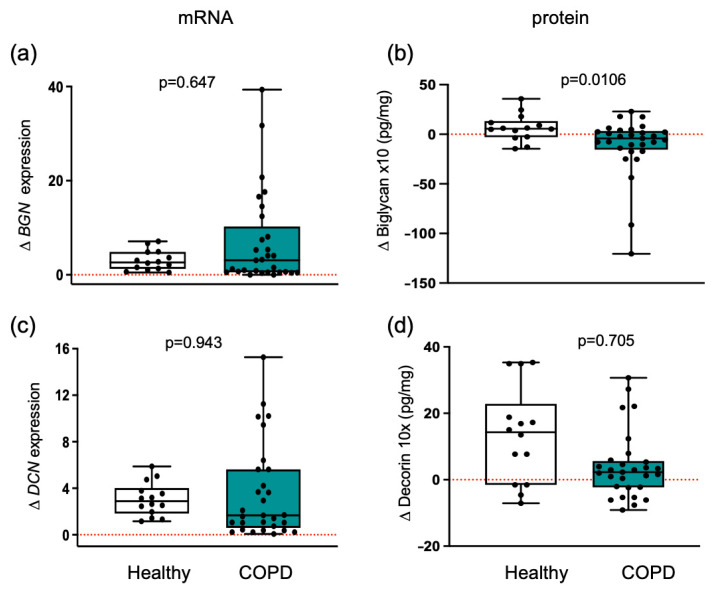
Training resulted in a protective increase in structural biglycan only in healthy individuals. Changes in healthy individuals and patients with COPD are shown. Boxplots depict medians (black line) with interquartile ranges for: (**a**) *BGN* mRNA expression and (**b**) biglycan protein levels; (**c**) *DCN* mRNA expression and (**d**) decorin protein levels. Results in plots a and c are fold change, and in plots b and d are absolute concentration changes. Individual participant values are shown as black data points. Values between groups were analyzed using a two-way repeated-measures ANCOVA (factors: group × time). Statistical significance is indicated in the graphs. The red dotted line indicates the level of no change with exercise training within each group.

Training elicited distinct proteoglycan responses in both groups. The biglycan protein response to training differed (*p* = 0.0106) between healthy participants and patients with COPD (Figure 3). Following training, biglycan levels increased by 6.5 pg/mL in healthy participants but decreased by 11.2 pg/mL in COPD patients, indicating altered muscle structural stability and impaired adaptation in COPD. Consistent with this interpretation, post-training changes in mean CSA demonstrated a significant inverse correlation with alterations in biglycan protein levels (r_S_ = −0.561, *p* = 0.010) across all participants. No significant differences in *BGN* mRNA expressions were observed between groups (Figure 3).

Decorin protein levels increased in response to training in both groups. Although decorin levels in healthy muscle were approximately 1.6-fold higher than in COPD muscle, this difference did not reach statistical significance (*p* = 0.705) (Figure 3). Changes in *DCN* mRNA expression were not significant between groups.

### 3.5. Matricellular Proteins Respond Differently to Training in Healthy and COPD Muscle

**Figure 4:**
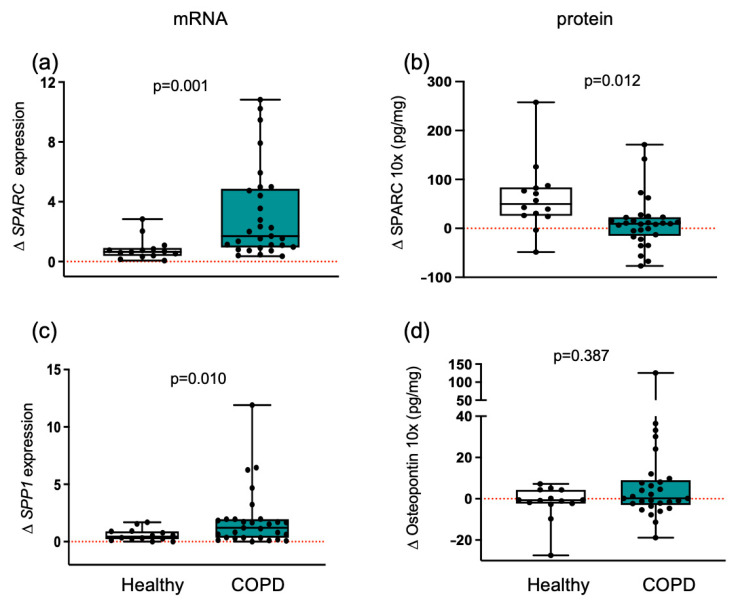
Training promoted an increase in the exerkine SPARC only in healthy individuals. Changes in healthy individuals and patients with COPD are shown. Boxplots depict medians (black line) with interquartile ranges for: (**a**) *SPARC* mRNA expression and (**b**) SPARC protein levels; (**c**) *SPP1* mRNA expression and (**d**) osteopontin protein levels. Results in plots a and c are fold change, and in plots b and d are absolute concentration changes. Individual participant values are shown as black data points. Values between groups were analyzed using a two-way repeated-measures ANCOVA (factors: group × time). Statistical significance is indicated in the graphs. The red dotted line indicates the level of no change with exercise training within each group.

Exercise training induced an increase (*p* = 0.012) in SPARC protein levels in healthy individuals (Figure 4). Elevated levels of this key exerkine suggest a more efficient response in healthy muscle compared with COPD patients. In contrast, osteopontin protein remained unchanged between groups. At the transcriptional level, training induced increases in *SPARC* (*p* = 0.001) and *SPP1* (*p* = 0.010) mRNA expression in muscle from COPD patients (Figure 4).

### 3.6. ECMs with Adhesive and De-Adhesive Properties

**Figure 5:**
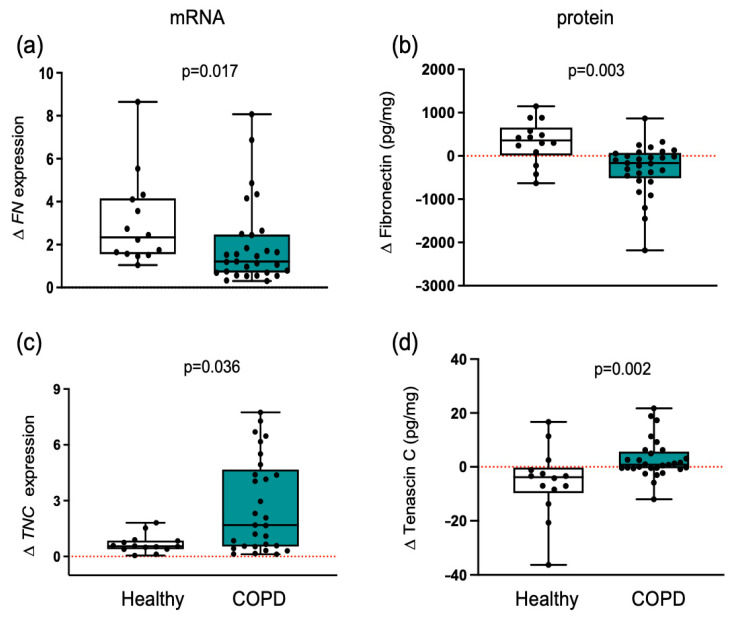
Training promotes an increase in ECM adhesive properties only in healthy individuals. Changes in healthy individuals and patients with COPD are shown. Boxplots depict medians (black line) with interquartile ranges for: (**a**) *FN* mRNA expression and (**b**) fibronectin protein levels; (**c**) *TNC* mRNA expression and (**d**) tenascin C protein levels. Individual participant values are shown as black data points. Results in plots a and c are fold change, and in plots b and d are absolute concentration changes. Values between groups were analyzed using a two-way repeated-measures ANCOVA (factors: group × time). Statistical significance is indicated in the graphs. The red dotted line indicates the level of no change with exercise training within each group.

Exercise training elicited divergent responses in muscle fibronectin protein levels (*p* = 0.003) between healthy individuals and patients with COPD (Figure 5). In healthy muscle, training increased significantly both fibronectin protein levels and *FN* gene expression, whereas in COPD muscle, training decreased fibronectin protein levels and reduced *FN* gene expression. Because cell adhesion to fibronectin can be inhibited by tenascin C, we also measured the expression levels of this antiadhesive protein [21].

Tenascin C is an anti-adhesive protein capable of disrupting focal adhesion and inhibiting integrin signaling. Opposing responses after training were also detected between groups (Figure 5). Exercise training promoted a divergent response in tenascin C protein between groups (*p* = 0.002). Training significantly decreased tenascin C protein levels and lowered *TNC* gene expression in healthy individuals, whereas in COPD patients, tenascin C protein levels and *TNC* gene expression were increased. The anti-adhesive properties of tenascin C protein may have contributed to altered muscle adaptation in COPD patients, as reduced adhesion of satellite cells to the ECM could impair their survival and supporting role in myofiber redistribution. In line with this interpretation, across all participants, post-training changes in tenascin C protein levels were significantly and inversely correlated with type IIx distribution (r_S_ = −0. 755, *p* = 0.001) and type I distribution (r_S_ = −0.46, *p* = 0.022).

In addition to the anti-adhesive properties of tenascin C, this protein is a marker of necroptosis [22], indicating that muscle from COPD patients may present necroptosis after training.

Given the opposing roles of fibronectin and tenascin C, we calculated their ratio. In healthy individuals, the fibronectin/tenascin C protein ratio increased nearly fivefold (from 48.0 pre-training to 237.7 post-training). In contrast, muscle from COPD patients showed a 2.5-fold reduction, with the ratio decreasing from 216.0 to 86.1.

In addition, absolute values for fold mRNA expression and protein levels are available in the online Supplementary Tables S1 and S2.

## 4. Discussion

Peripheral muscle wasting and impaired regeneration are commonly observed in patients with COPD [1,14]. Several etiologies underlie the loss of contractile myofiber in COPD, including disuse, hypoxemia, protein anabolism/catabolism imbalance, oxidative stress, local inflammation, and impaired regenerative capacity [2]. These unfavorable mechanisms contribute to the limitation of exercise capacity, a most debilitating feature in patients with COPD. While there is a decrease in functional contractile tissue, the relative area occupied by non-contractile intramuscular connective tissue and its extracellular matrix (ECM) proportionally increases. Aging of human skeletal muscle is associated with increased passive stiffness, but it is still debated whether this increased ECM is a determinant of the resistance to training-induced muscle adaptation [4]. In response to exercise training, this study compares the changes in muscle fiber phenotype and the level of ECM markers in COPD patients and healthy young individuals to provide insight into how normal adaptive processes differ under conditions of chronic disease. The training-induced ECM adaptation in healthy young individuals promotes changes in the interstitial cell environment (cell niche) conducive to muscle growth and repair, whereas diversion from this profile may not be particularly beneficial to muscle adaptation despite adequate exercise training program.

Consistent with previous reports, a higher proportion of type II myofibers, particularly the type IIa subtype, was observed in muscle from COPD patients compared with healthy individuals, both before and after training [1,14]. The prescribed exercise intervention effectively increased overall mean fiber CSA in both groups. Increased gain in capillary to fiber ratio in COPD patients compared to healthy young confirms previous studies demonstrating that the magnitude of improvement is greater in deconditioned individuals with less preserved capillary network at the outset of the study [23]. Our findings show implications of post-training changes in specific structural and anti-adhesive ECM proteins, with the magnitude of changes in mean fiber CSA (collagen IV, biglycan) and fiber type I and type IIx redistribution (tenacin C). However, significant hypertrophy of type I fibers was observed only in healthy individuals, whereas COPD patients exhibited pronounced adaptive responses primarily within type II fibers [3]. This divergence may reflect disease-related alterations in oxidative capacity, mitochondrial function, neuromuscular junction activation, or impaired extracellular matrix remodeling, all of which could constrain type I fiber adaptation in COPD [3]. In addition, systemic inflammation and muscle wasting, commonly associated with COPD, may further bias the adaptive response toward type II fibers. Together, these findings support the concept that while exercise remains beneficial across populations, the mechanisms driving muscle plasticity differ fundamentally between healthy and diseased states.

Recent studies give evidence of the critical role of satellite cells in muscle plasticity and hypertrophic adaptation [17,18,24]. PAX7 and MYOD1 are specific satellite cell transcription factors and essential regulators of the myogenic process. PAX7 is a marker of quiescent satellite cells within adult skeletal muscle, whereas MYOD1 is a marker of activation and differentiation of these cells [19]. In the present study, the similar ratio of *PAX7* to *MYOD1* gene expression between groups indicates that exercise training elicited comparable satellite cell commitment, activation, and differentiation in both COPD patients and healthy individuals. This finding prompted us to investigate a possible downstream event responsible for the differences in muscle adaptation. Furthermore, there is evidence that muscle stretching is likely to be beneficial to align the connective tissue and reduce muscle inflammation as stretch deforms the ECM independently of muscle activation [25]. Further studies testing the effect of stretch exercise on ECM modulation have shown that it reduces the expression of TGF-B1, which would translate into lower intramuscular fibrosis [26].

The two participant groups displayed contrasting responses regarding ECM components involved in adhesion. In COPD patients, training was associated with a deficit in ECM adhesive capacity, as evidenced by decreased fibronectin and increased tenascin C levels. Although training effectively activated and differentiated satellite cells in COPD muscle, the impaired ECM adhesion likely compromised myonuclear accretion. Following muscle exposure to mechanical loading, satellite cells differentiate and fuse with existing myofibers to donate their nucleus to support fiber repair and adaptation [18]. The donation of nuclei is believed to occur during the repair of damaged myofibers and during times of heightened transcriptional activity, such as when the volume of a myofiber increases (e.g., fiber CSA). Myonuclear accretion relies on the adhesive properties of the ECM. Specifically, fibronectin provides both an adhesive and directional cue for migrating myoblasts [27]. Following training, COPD patients presented lower levels of fibronectin, which wouldn’t be supportive of satellite cell adhesion [27,28]. Consequently, differentiated satellite cells (myoblasts) undergo anoikis, a form of programmed cell death that occurs when cells lose their attachment to the ECM [27,28]. Therefore, in COPD patients, differentiated satellite cells may thus be more susceptible to anoikis, limiting muscle regeneration and growth. In addition, fibronectin plays a role in the fusion of new muscle fibers and integration into existing tissue [27]. In contrast, in muscle from healthy individuals, the ECM adhesive capacity was enhanced by increased levels of fibronectin and lower tenascin C, supporting myonuclear accretion and promoting muscle hypertrophy. Furthermore, high levels of tenascin C are indicative of myofiber necroapoptotic events in muscle from COPD patients.

Reduced ECM adhesion may also contribute to the preferential hypertrophy of type II fibers in COPD muscle. Moro et al. [24] demonstrated that type I and type II fibers rely on distinct hypertrophy mechanisms. Myonuclear accretion from satellite cells is essential for maintaining a stable cytoplasmic-to-myonuclear domain ratio during hypertrophy. Type II fibers can enlarge their myonuclear domain with minimal addition of new nuclei, whereas type I fiber hypertrophy is more strictly dependent on substantial myonuclear accretion. In healthy individuals, coordinated changes in ECM composition and satellite cell dynamics likely underpin the observed type I fiber hypertrophy [24]. In contrast, in COPD, impaired ECM adhesion may restrict myonuclear accretion, creating an unfavorable condition for type I fiber hypertrophy. Together, these findings highlight the interdependence of ECM regulation and satellite cell activity as critical determinants of muscle plasticity in both healthy and COPD patients.

These differences in ECM adhesion may be further influenced by integrin-mediated signaling. Integrins connect satellite cells to the ECM, and they are critical receptors for mechanotransduction and for promoting cell survival and fusion during muscle growth. Both groups show a similar change in *ITGB1* expression following training, which indicates that the levels of integrin B1 may not play a role in the differential adaptation between groups.

The other ECM component differentially affected by training between groups was collagen IV, a protein that forms a scaffolding network at the basement membrane, integrating the ECM micro-environment and cell signaling. In addition, collagen IV binds and sequesters growth factors, therefore regulating muscle adaptation response to training [5,6]. Although training induced similar *COL4A1* gene expression in both groups, collagen type IV protein levels decreased after training only in healthy individuals, making it more efficient for mechano-transduction and signaling [5]. Efficient collagen IV turnover in healthy individuals will likely alter the satellite cell niche, facilitating its migration through the intramuscular connective tissue, as well as the release of growth factors that can facilitate muscle adaptation [29]. At the same time, the increase in collagen IV protein would increase the thickness of the basement membrane in COPD patients, restricting the diffusion rate for gas and metabolic substrate reaching muscle fibers. This may explain the greater increase in mean fiber CSA seen in healthy individuals and supported by an inverse association between post-training changes in mean fiber CSA and collagen IV across all studied participants. In addition, we observed an inverse association between the training-induced changes in type IIx and collagen IV across all study participants. The discrepancies between ECM mRNA expression and protein levels are likely a result of the deficient ECM protein turnover characteristic of aged skeletal muscle, increasing the susceptibility to deleterious posttranslational modifications and increased stiffness [29]. Whereas, in healthy individuals, training may have resulted in an increased turnover of collagen to allow the reorganization of tissue, and a more prolonged training would be needed to observe an increase in protein levels [29,30].

A limitation of this study is that all participants were male; including female participants would clarify whether the findings extend to the broader COPD population. COPD patients were not matched for comorbidity, nutritional status, and physical activity. A study including a larger number of patients is necessary to allow patient stratification for ECM analyses. Since the mean CSA in healthy individuals was significantly higher than in COPD patients, normalizing the ELISA results to myosin heavy chain rather than total protein would have provided a more accurate reflection of changes in ECM levels relative to the increase in contractile muscle protein. Furthermore, direct measurements of satellite-cell abundance or activation (e.g., via immunohistochemistry), fusion (e.g., myomaker/myomerger), apoptosis/necroptosis markers, or myonuclear counts were not performed. Impaired myonuclear accretion and increased necroptosis are inferred from ECM patterns, specifically fibronectin and tenascin C. Fibronectin supports adherence of muscle-cell precursors, which is essential for cell survival, differentiation, and hypertrophic myonuclear accretion. Conversely, tenascin C is an anti-adhesive protein and inhibits fibronectin adhesive properties, potentially disrupting satellite cell adhesion to the ECM. In addition, we acknowledge the issue of age as a potential major contributing factor to the response in exercise training as control subjects were much younger than COPD patients. Engaging young healthy men as controls (instead of age-matched individuals) poses a significant limitation, given that the ECM turnover is age-sensitive, and thus differences observed may partly reflect aging rather than COPD per se.

## 5. Conclusions

In patients with COPD, exercise training elicited distinct alterations in ECM protein expression associated with tissue structure and integrity, adhesion capacity, myogenesis, and necroptosis, relative to healthy participants. These results indicate that the attenuated training adaptations frequently observed in COPD may, at least in part, reflect impaired intramuscular ECM remodeling.

## Figures and Tables

**Table 1:** Demographic and lung function characteristics of healthy individuals and COPD patients.

Characteristics	Healthy	COPD
Individuals (*n*)	14	29
Age (years)	21.80 ± 0.60	65.68 ± 1.42 ^†^
Weight (kg)	74.20 ± 2.10	72.89 ± 2.62
BMI (kg m^−2^)	23.45 ± 0.58	25.94 ± 0.80
FFM index (kg m^−2^)	23.70 ± 0.67	17.76 ± 0.40
FEV_1_ (L)	−	1.13 ± 0.08
FEV_1_ (% predicted)	−	43.32 ± 3.29

Data are presented as mean ± SEM. BMI: body mass index; FFM: fat-free mass; FEV_1_: forced expiratory volume in 1 s; FVC: forced vital capacity; † *p* < 0.05 significance level between COPD and healthy groups.

## Data Availability

All relevant data are included in the manuscript, and they are part of the EK thesis submitted for the doctorate degree at Northumbria University, Newcastle. Any additional information may be obtained by contacting the corresponding author.

## Supplementary Materials

The following supporting information can be downloaded at: https://www.mdpi.com/article/10.3390/cells14211656/s1. Figure S1: *COL1A1* fold gene expression does not change between groups. Changes in healthy individuals and patients with COPD are shown. Boxplots depict medians (black line) with interquartile ranges for *COL1A2* mRNA expression. Results are in mRNA fold change. Values between groups were analyzed using a two-way repeated-measures ANCOVA (factors: group × time). The level of significance is indicated in the graph. The red dotted line indicates the level of no change with exercise training within each group; Table S1: Mean fold mRNA expression of ECM molecules before and after training; Table S2: Mean ECM protein levels before and after training.

## Abbreviations

The following abbreviations are used in this manuscript:

BGN	Byglican
COL1	Collagen type I
COL4	Collagen type IV
CSA	Cross-sectional area
DCN	Decorin
ECM	Extracellular matrix
FEV1	Forced expiratory volume in 1 s
FN	Fibronectin
FVC	Forced vital capacity
GAPDH	glyceraldehyde 3-phosphate dehydrogenase
MRF	Myogenic regulatory factor
MYOD1	myogenic differentiation 1
Pax7	Paired box 7 gene
TNC	Tenascin C
