# Supplementary material for: Distinct Intramuscular Extracellular Matrix Protein Responses to Exercise Training in COPD and Healthy Adults and Their Association with Muscle Remodeling
Source: Cells. 2025 Oct 22;14(21):1656. doi: 10.3390/cells14211656 (PMC12607679; doi:10.3390/cells14211656)
Supplement: Supplementary file 1 [file cells-14-01656-s001.zip › cells-3881249-supplementary.pdf]

Supplementary Table S1. Mean fold mRNA expression of ECM molecules before and after training

ECM proteins	Healthy		COPD	
	Pre-training	Post-training	Pre-training	Post-training
COL1A1	1.2± 0.5	15.9 ± 2.9*	5.5 ± 1.8	14.8 ± 3.5**
COL1A2	3.1± 0.8	5.9 ± 1.3*	0.9 ± 0.3	2.3 ± 0.4***
COL4A1	1.2 ± 0.5***	15 ± 2.9	12.5 ± 2.5	32.4 ± 5.8**
BGN	1.4 ± 0.4	1.7 ± 0.3	2.3 ± 0.5	6.3 ± 1.2***
DCN	1.2 ± 0.3	2.5 ± 0.6*	0.8 ± 0.1	1.6 ± 0.3**
SPARC	1.2 ± 0.4	2.6 ± 0.8	0.6 ± 0.5	2.2 ± 1.9
SPP1	0.7 ± 0.3	0.8 ± 0.3	0.4 ± 0.2	0.9 ± 1.2
FN	1.2 ± 0.7	3.5 ± 0.7*	2.7 ± 0.5	3.0 ± 0.7
TNC	0.6 ± 0.2	2.8 ± 1.0	1.3 ± 0.3	6.4 ± 1.7*
PAX7	2.1 ± 0.6	4.4 ± 0.5**	9.6 ± 2.6	12.1 ± 5.5*
MYOD1	1.6 ± 0.4	3.0 ± 0.8	2.5 ± 0.9	3.3 ± 1.2
MSTN	1.0 ± 0.5	1.6 ± 0.9	2.0 ± 0.3	2.3 ± 0.4
ITGB1	1.5 ± 0.5	1.8 ± 0.6	1.5 ± 0.5	2.2 ± 0.6

Data are presented as mean ± SEM. Three experimental replicates per individual muscle sample. *$p < 0.05$, **$p<0.01$ and ***$p<0.0001$ significance within a group analyzed using statistical Wilcoxon test

Supplementary Table S2. Mean ECM protein levels before and after training

ECM proteins	Healthy		COPD	
	Pre-training	Post-training	Pre-training	Post-training
Collagen I 10x (pg/mg)	888.5 ± 276.9	289.2 ± 73.2**	36.41 ± 3.4	36.41 ± 3.9
Collagen IV 10x (pg/mg)	48.36 ± 6.0	37.37 ± 4.3*	41.92 ± 3.9	41.92 ± 4.2**
Biglycan 10x (pg/mg)	17.77 ± 2.0	17.77 ± 3.4	17.77 ± 5.3	19.02 ± 2.8
Decorin 10x (pg/mg)	58.77 ± 3.8	72.15 ± 6.1	36.43 ± 1.4	36.43 ± 2.3*
SPARC 10x (pg/mg)	118.1 ± 7.7	184.5 ± 13.4**	155.8 ± 8.0	167 ± 9.9
Osteopontin 10x (pg/mg)	6.403 ± 2.7	4.57 ± 0.82	12.39 ± 2.8	20.62 ± 6.1
Fibronectin (pg/mg)	466.9 ± 72.4	786 ± 84.9*	1178 ± 160.4	886.5 ± 150.1**
Tenascin C 1 (pg/mg)	16.57 ± 2.8	10.95 ± 1.5	10.81 ± 1.3	13.24 ± 1.3

Data are presented as mean ± SEM. Three experimental replicates per individual muscle sample. *$p < 0.05$ and **$p<0.01$ significance within a group analyzed using statistical Wilcoxon test

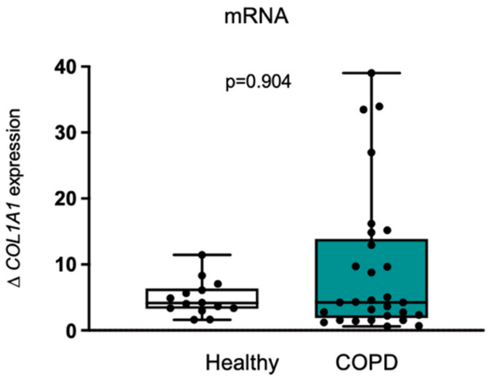

Supplementary Figure S1. *COL1A1* fold gene expression does not change between groups. Changes in healthy individuals and patients with COPD are shown. Boxplots depict medians (black line) with interquartile ranges for *COL1A2* mRNA expression. Results are in mRNA fold change. Values between groups were analyzed using a two-way repeated-measures ANCOVA (factors: group × time). The level of significance is indicated in the graph. The red dotted line to indicate the level of no change with exercise training within each group.
